# The Potential Gastro-Protective Effect of Qaysum (Achillea fragrantissima) Against Ethanol-Induced Gastric Ulcer in Wistar Albino Rats

**DOI:** 10.7759/cureus.47696

**Published:** 2023-10-26

**Authors:** Thekra B Alhejaily, Zaenah Z Alamri, Fatima S ALaryani, Suhayla H Shareef

**Affiliations:** 1 Department of Biology, College of Science, University of Jeddah, Jeddah, SAU; 2 Department of Biology, Salahaddin University-Erbil, Erbil, IRQ

**Keywords:** rats, oxidative stress, protective effects, gastric ulcer, achillea fragrantissima extract

## Abstract

Gastric ulcer (GU) is among the peak prevalent syndromes. This study investigates the defensive properties of *Achillea fragrantissima* (Forssk.) Sch.Bip. extract (AFE) against ethanol-induced stomach lesions. Twenty-eight rats were allocated into negative control, positive control, AFE + ethanol, and omeprazole ethanol. In serum and gastric homogenates, oxidative stress displays (e.g., malondialdehyde (MDA), decreased glutathione (GSH), superoxide dismutase enzyme (SOD), and catalase enzyme (CAT)) and inflammatory parameters (e.g., tumor necrosis factor-alpha (TNF-α)) were estimated. GU markers (gastric lesions, ulcer index (UI), pH) were evaluated, and gastric histopathological examinations were performed. The positive control cluster exhibited severe gastric mucosal injuries, reduced stomach mucus secretion, and pH of gastric content. Furthermore, AFE-pretreated rats displayed meaningfully increased periodic acid-Schiff (PAS) countenance in their stomach epithelial layers. Pretreatment with AFE reduced stomach lesions, UI, MDA, and TNF-α levels, while mucus, pH, CAT, GSH, and SOD levels increased. Stomach examination showed significant improvement in gastric mucosa reduced edema and leukocyte infiltration of the submucosal level in pretreatment with the AFE and omeprazole groups versus the ethanol group. Additionally, AFE extracts increase the intensity of the stomach epithelium's PAS. The acute toxicity experiment with an advanced dosage of 5 g/kg AFE did not exhibit any signs of toxicity in the rats. In conclusion, the AFE reduced the effect of ethanol on the gastric mucosa, which may be due to its antioxidants and anti-inflammatory properties.

## Introduction

The prevention or cure of gastric ulcer (GU) is one of the most urgent problems facing medicine today. The inequity among hostile and defensive factors in the gastric epithelial fence leads to stomach ulceration. Gastric ulceration is caused by many risk factors, including gastric hydrochloric acid (HCl), oxygen free radicals, ethanol, *Helicobacter pylori* (*H. pylori*), and the use of non-steroidal anti-inflammatory drugs (NSAIDs) [1].

Ethanol-induced GU model is useful for investigating the efficacy of new medications or testing substances with cytoprotective properties. Ethanol produces GU through various mechanisms, such as increased secretion of gastric histamine, which leads to HCl release from parietal cells, increased construction of prooxidative and proinflammatory enzymes, and free O_2_ radicals, subsequent in oxidative pressure and apoptosis. This is due to the invasion of activated neutrophils into the injury site, which causes stomach mucosa damage. Moreover, ethanol reduces nitric oxide (NO) levels required for gastric mucosa physiological activity, leading to a decline in gastric blood flow and creating hemorrhagic lesions that can solubilize gastric mucus constituents [2].

Management of GU has become a challenge worldwide. While various treatments are available, the primary approach is to reduce gastric acid output, acid neutralization, and increase antioxidant levels in the stomach [2]. Medications, for example, proton pump inhibitors (PPIs), histamine (H)2-receptor antagonists, and muscarinic (M)-1-receptor blockers, are used to treat GU. However, these drugs have adverse side effects, for example, gynecomastia, arrhythmia, thrombocytopenia, and enteric infections [3]. As a result, herbal medications are now being considered a viable alternative therapy for protecting and treating GU due to their lower cost, perceived efficacy, availability, and little or no adverse effects [4,5].

*Achillea fragrantissima* extract (AFE), also known as "Qaysum, Gesoom, Bu`eithraan" in Arabic and "Lavender cotton, Grade robe" in English, is a flowering plant belonging to the genus *Achillea L.* (yarrow) of the Asteraceae family. It is traditionally used for treating various ailments, such as hepatobiliary disorders, hypertension, respiratory diseases, skin inflammations, gastrointestinal disorders, stomach aches, diabetes mellitus, and wound healing. The AFE possesses excellent antioxidant, anti-inflammatory, anti-microbial, antiviral, and anthelmintic properties due to its rich flavonoids, polyphenols, terpenes, and alkamides [6,7].

This experimental study aimed to examine the effects of ethanol-provoked GU in adult male albino rats on oxidative stress markers and stomach structure. Additionally, the potential defensive properties of oral administration of AFE against ethanol-convinced GU and the possible mechanisms of action were investigated.

## Materials and methods

Acute toxicity study

Thirty-six Wistar albino healthy rats (21-28 days old, weights of 180-250 g) were used in this study. An acute poisonousness test was applied to decide a nontoxic dose for the AFE. Rats were distributed equally into three clusters: vehicle (normal saline, 5 mL/kg), 2,000 mg/kg, and 5,000 mg/kg AFE (5 mL/kg). Previous supplementation and dealing, 24 hours starving was applied to altogether rat groups (only food, not water). Nourishment was detached for around three to four hours after being fed with the AFE. The rats' practices one day to two days afterward the gavage AFE were checked for the start of controlled or toxicological symbols. The mortality rate was calculated for over 14 days. Rats experienced high-dose injections of xylazine and ketamine anesthesia on the 15th day. Blood collected from intracardial punctures was divided into serum samples taken for biochemical examination, kidney, and liver for histological assessment.

Chemicals

AFE

To obtain the AFE, the stems, and leaves of *A. fragrantissima* were purchased from a local market and dried at room temperature for one week, away from sunlight. After drying, plant materials were ground using an electric mill; 500 g of the resulting powder was placed in a flask with 2 L of 70% ethanol and mixed. Then, the mixture was left to stand for 72 hours. After that, it was filtered, located in a flat container, and dehydrated at 25 °C. The dry excerpt is kept at 4^ °^C until used. To administer the AFE, 4 g of the extract was dissolved in 20 mL of distilled water [8].

Omeprazole

Omeprazole was acquired from a local pharmacy and dissolved in distilled water to get a concentration of 20 mg/kg [9]. Two omeprazole capsules were opened and crushed, and the resulting powder was dissolved in 10 ml of distilled water. Each rat was administered 1 ml of solution.

Animals

Ethical Approval

This research was accepted by the Ethical Committee of the Faculty of Pharmacy, King Abdel-Aziz University, Jeddah, Saudi Arabia (approval # PH-1444-34, 7/2/2023). The rats were treated in accordance with the Animal Research Reporting of In Vivo Experiments (ARRIVE) guidelines.

Twenty-eight healthy Wistar albino male rats, weighing 180-250 g, were used in this research. The rodents were acquired from the College of Pharmacy, King Abdulaziz University, Jeddah, Saudi Arabia. The rats were housed in plastic cages (individual animals) at room temperature and 70% humidity, for 12 hours light and 12 hours dark cycle, and had free admission to standard rodent diet and water.

Induction of GU

All animals were fasted for 24 hours before C2H5OH administration, except for water, to eliminate exogenous nutritive effects. The induction was performed by oral administration of 96% ethanol (1 mL/kg) by intra-gastric gavage [10]. Rats were killed one hour later by cervical dislocation after being anesthetized.

Experimental design

Experimental animals were separated into four collections (seven rats each) as follows:

(1) Negative control group (G1): rats fed orally normal saline (5 mL/kg) for 21 days

(2) Positive control group (G2): rats fed 5 mL/kg normal saline and then administered 96% ethanol (1 mL/kg) on day 22

(3) AFE + ethanol group (G3): rats treated orally with AFE (800 mg/kg) [8] for 21 days before ethanol administration

(4) Ethanol + omeprazole group (G4): rats received omeprazole orally (20 mg/kg) for 21 days before ethanol administration.

The total weight of the body was recorded at the beginning and end of the experiment using a digital balance.

Determination of gastric pH

Stomachs were cut along the bigger curving portion, and stomach juice was accumulated, centrifuged, and examined to quantity the pH acidity of gastric fluid from the supernatant using 0.1 N NaOH solution, and gastric juice pH was determined using a pH 1-14 test paper.

Blood sample collection

Blood specimens were acquired from veins of retro-orbital into a serum tube at the experimental end. Blood was centrifuged at 3,000 rpm/5 min at 4 °C to get the serum that aliquots and stored at -20 °C till used.

Gastric homogenate collection

Gland sections of stomach tissue were removed and cleaned with ice-cold normal saline. Consuming a homogenizer, half of the gastric was homogenized in ice-cold phosphate-buffered saline (0.1 mol/L), including a protease inhibitor cocktail of mammalian [11]. The mixtures were centrifuged at 4,500 rpm/15 minutes at 4 °C, and supernatants were collected for further analysis of biochemical parameters. The collected gastric homogenate supernatant was stored at -80 °C until used.

Biochemical analysis

Serum and gastric homogenate tissue quantities of malondialdehyde (MDA), glutathione (GSH), superoxide dismutase enzyme activity (SOD), catalase enzyme activity (CAT), and inflammatory parameters as tumor necrosis factor-alpha (TNF-α) using specific ELISA kits.

Gross examination of gastric mucosa

The animals were anesthetized, one hour after ethanol administration. The stomach was unlocked, and the pylorus was tight to gather stomach liquid. The animals' gut was detached, opened along the bigger curvature, and cleaned with 0.9% cold saline solution. A macroscopic gastric examination was performed to detect any hemorrhagic lesions on the glandular mucosa.

Measurement of ulcer index (UI) and protective index (PI)

UI was designed by the formula: UI=10X (total ulcerated zone/total mucosal zone), and PI was counted by equation PI= ((UI of ethanol-UI of drug-treated)/UI of ethanol) × 100 [12].

Tissue preparation for light microscopy

Stomach samples were divided into two sections, one for histopathological examination and the other for gastric homogenate preparation for biochemical analyses. The tissues designated for histopathological examination were fixed in 10% formalin and then processed using the paraffin technique. Subsequently, 5-6 μm slices were prepared and discolored with hematoxylin and eosin (H&E) for visualization by an ordinary light microscope to assess the general histological structure and detect any pathological changes.

Periodic acid-Schiff (PAS) dye

The mucus content in the stomach epithelium was identified by discoloration of a 5 μm sliced stomach layer with the PAS pigment to evaluate the glycoprotein (low or high pH) of the stomach mucus according to the producer’s manufacture (Sigma Diagnostics™ Periodic Acid Schiff Kit (PAS) Kit, Merk, Germany). Photomicrograph was obtained by ImageJ software (US National Institutes of Health, Bethesda, MD).

Statistical analysis

Standards were articulated by way of mean +/- standard deviation (SD). Data were evaluated using Statistical Product and Service Solutions (SPSS) (version 22; IBM SPSS, IBM Corp., Armonk, NY). The Shapiro-Wilk rest the checked data distribution normality. The data were analyzed using the one-way ANOVA test shadowed by Tukey's test for comparison between groups in data distributed normally, and the Kruskal-Wallis shadowed by the Mann-Whitney test for abnormally distributed data such as pH and UI. A P < 0.05 was statistically significant.

## Results

Acute toxicity test

**Figure 1:**
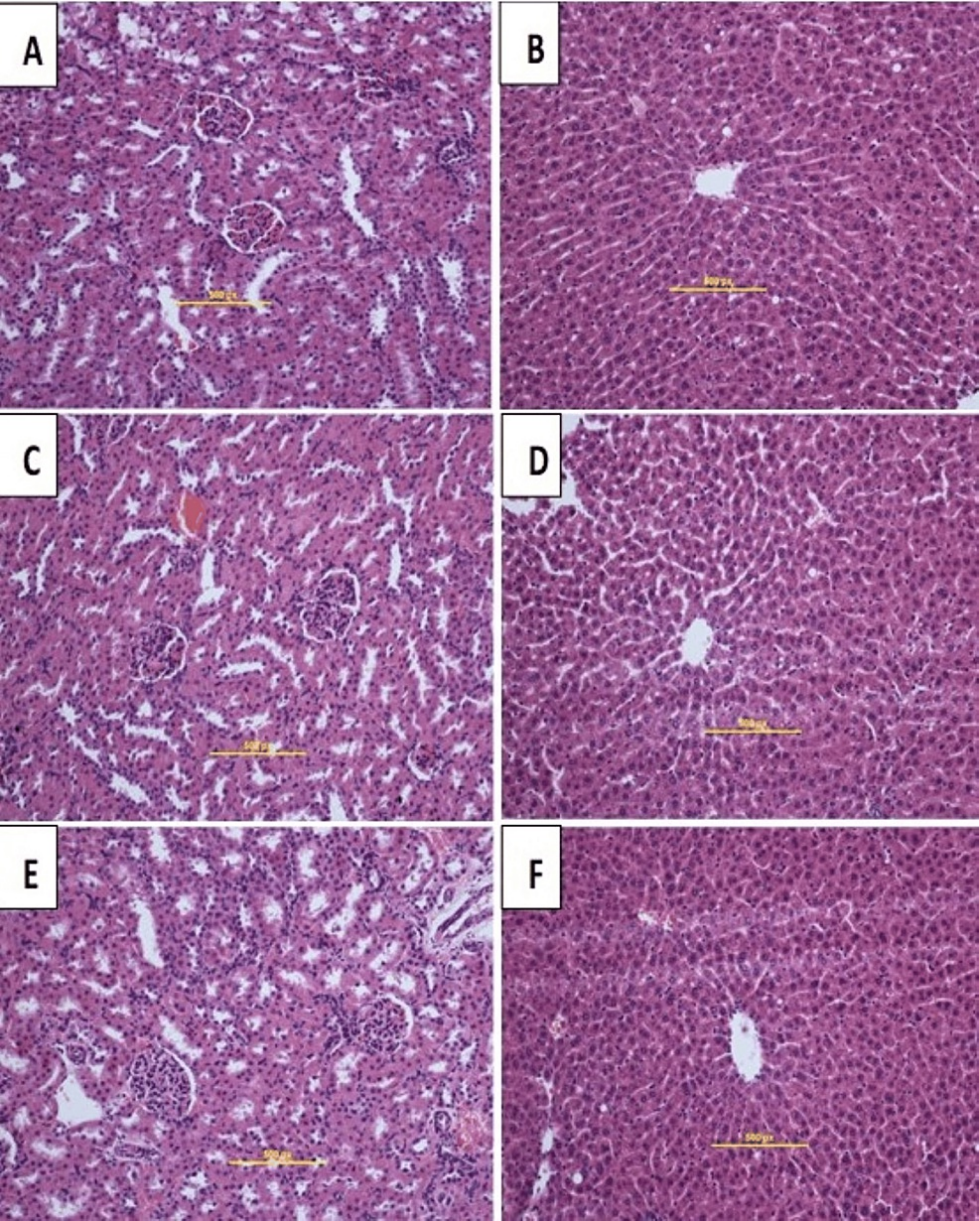
Histological slices of the kidney (left column) and liver (right column) in the acute poisonousness test. Rats fed with 5 mL/kg vehicle (0.5% CMC) (A, B); rats fed orally with 2,000 mg/kg (2 mL/kg) (C, D); rats fed with 5,000 mg/kg (5 mL/kg) (E, F). No significant modifications were shown in the structures of livers and kidneys between the therapeutic group and control groups (H&E staining, 40×). Histological slices of the kidney (left column) and liver (right column) in the acute poisonousness test. Rats fed with 5 mL/kg vehicle (0.5% CMC) (A, B); rats fed orally with 2,000 mg/kg (2 mL/kg) (C, D); rats fed with 5,000 mg/kg (5 mL/kg) (E, F). No significant modifications were shown in the structures of livers and kidneys between the therapeutic group and control groups (H&E staining, 40×).

The acute toxicity assessment was done on 36 rats and separated equally into three collections: group 1 (G1), as a vehicle (normal saline, 5 mL/kg); group 2 (G2), fed one dose of 2,000 mg/kg; and group 3 (G3), fed one dose of 5,000 mg/kg AFE. Rats were reserved under surveillance for approximately half a month. The investigation outcomes found that altogether rats lived, and none practiced any significant observable signs of poisonousness at these doses. Moreover, continuous remarks showed no abnormal symbols and symptoms, and no alterations in the behavior, body weight, or water and feed eating of the rats. The acute toxicity experiment did not exhibit any signs of toxicity. There was no histopathological sign of liver or kidney poisonousness (Figure 1). Additionally, the biochemistry examination of blood seemed normal (data not presented, but are available upon demand).

Body weights

The weight gains and percentage changes in weight gain were significantly increased in G3 (AFE + ethanol), versus G1 and G2, but were significantly decreased in G4 (omeprazole + ethanol) versus G1 and G2 (P < 0.0001 for both) (Table 1).

**Table 1:** The effect of qaysum (AFE) against ethanol-induced stomach ulcer on body weights in mature healthy male rats. *: significance vs. G1 (negative control), #: Significance vs. G2 (positive control). *: P < 0.05, ***: P < 0.001.

Experimental groups	Initial body weight (grams)	Final body weight (grams)	Weight gain (grams)	Change of weight gain (%)
G1 (negative control)	189.36±5.65	198.29±5.41	8.93±2.35	4.72±1.32
G2 (positive control)	195.29±11.25	203.57±11.16	7.79±2.40	4.12±1.09
G3 (AFE + ethanol)	189.43±7.83	203.57±4.79	14.14±3.44^*,##^	7.52±2.08^*,##^
G4 (omeprazole + ethanol)	199.00±3.49	198.57±4.76	-0.43±3.51^***,##^^#^	-0.22±1.78^***,##^^#^

UI, PI, and pH

The UI was significantly increased in G2 (positive control) and G3 (AFE + ethanol) versus G1 (negative control) (P < 0.0001, P < 0.0001, and P < 0.010, respectively) but was significantly decreased in G4 (omeprazole + ethanol) versus G2 (P < 0.0001). The levels of pH significantly decreased in G2 (positive control) versus G1 (negative control). However, there were significant increases in the level of PH in G3 (AFE + ethanol) and G4 (omeprazole + ethanol) (P < 0.0001, P < 0.010, P < 0.010, P < 0.010, and P < 0.010, respectively) (Table 2). The protective indices in G1, G2, G3, and G4 were 0, 20.95, 94.47, and 98.02, respectively.

**Table 2:** The effect of qaysum (AFE) against ethanol-induced gastric ulcer on the ulcer index (%) and pH in adult male rats. *: significance vs. G1 (negative control), #: Significance vs. G2 (positive control). *: P < 0.05, ***: P < 0.001. Significance was made by the Mann-Whitney test as data were not normally distributed.

Experimental groups	Ulcer index (%)	pH
G1 (negative control)	0.00±0.00	5.29±1.11
G2 (positive control)	2.53±1.37^***^	2.43±0.79^***^
G3 (AFE + ethanol)	2.00±1.72^***^	4.00±0.82^##^
G4 (omeprazole + ethanol)	0.05±0.07^###^	5.14±1.35^##^

Oxidative stress and inflammatory markers

The MDA levels in serum and gastric tissue homogenate were expressively increased in G2 (positive control) versus G1 (negative control), G3 (AFE + ethanol), and G4 (omeprazole + ethanol) (P < 0.0001 for all). CAT, GSH, and SOD levels in serum and gastric tissue homogenate were meaningfully decreased in G2 (positive control) versus G1 (negative control), G3 (AFE + ethanol), and G4 (omeprazole + ethanol) (P < 0.0001 for all) (Table 3). TNF-α levels in serum and gastric tissue homogenate were considerably increased in G2 (positive control) versus G1 (negative control), G3 (AFE + ethanol), and G4 (omeprazole + ethanol) (P < 0.0001 for all). The serum levels of TNF-α were meaningfully increased in G3 and G4 versus G1 (P < 0.050 and P < 0.010) (Table 4).

**Table 3:** The effect of qaysum (AFE) against ethanol-influenced stomach ulcer on the levels of oxidative stress markers in serum and gastric homogenate in adult male rats. MDA: Malonaldehyde, CAT: Catalase, GSH: Glutathione, SOD: Superoxide dismutase. *: significance vs. G1 (negative control), #: Significance versus G2 (positive control). *: P < 0.05, ***: P < 0.001.

Experimental groups	MDA		CAT		GSH		SOD	
	Serum (nmol/ml)	homogenate (nmol/g)	Serum (ng/ml)	homogenate (ng/g)	Serum (µmol/L)	Homogenate (ng/g)	Serum (ng/ml)	homogenate (ng/g)
G1 (negative control)	1.70±0.26	1.44±0.4512	121.21±16.26	125.97±19.22	608.15±100.45	618.32±118.93	3.97±0.78	4.41±0.52
G2 (positive control)	3.14±0.75^***^	2.80±0.49^***^	47.06±6.86^***^	30.78±7.53^***^	237.33±36.42^***^	428.71±52.57^**^	1.26±0.21^***^	1.36±0.21^***^
G3 (AFE + ethanol)	2.25±0.29^###^	1.82±0.60^###^	110.88±15.74^###^	137.74±7.23^###^	622.26±144.83^###^	729.48±77.60^###^	3.83±0.60^###^	3.85±0.32^###^
G4 (omeprazole + ethanol)	2.13±0.22^###^	1.64±0.29^###^	128.26±11.19^###^	124.59±5.60^###^	605.35±92.70^###^	743.05±107.05^###^	3.85±0.41^###^	4.50±0.43^###^

**Table 4:** The effect of qaysum (AFE) against ethanol-induced stomach ulcer of TNF-α levels in serum and gastric homogenate in adult male rats. *: significance vs. G1 (negative control), #: Significance vs. G2 (positive control). *: P < 0.05, ***: P < 0.001.

Experimental groups	Serum TNF-α (mg/L)	Gastric homogenate TNF-α (ng/g) protein
G1 (negative control)	198.60±11.96	241.14±20.71
G2 (positive control)	429.03±18.57^***^	483.41±27.07^***^
G3 (AFE + ethanol)	264.82±70.87^*,###^	268.67±32.42^###^
G4 (omeprazole + ethanol)	273.70±36.68^**,###^	254.25±34.62^###^

Morphological examination

**Figure 2:**
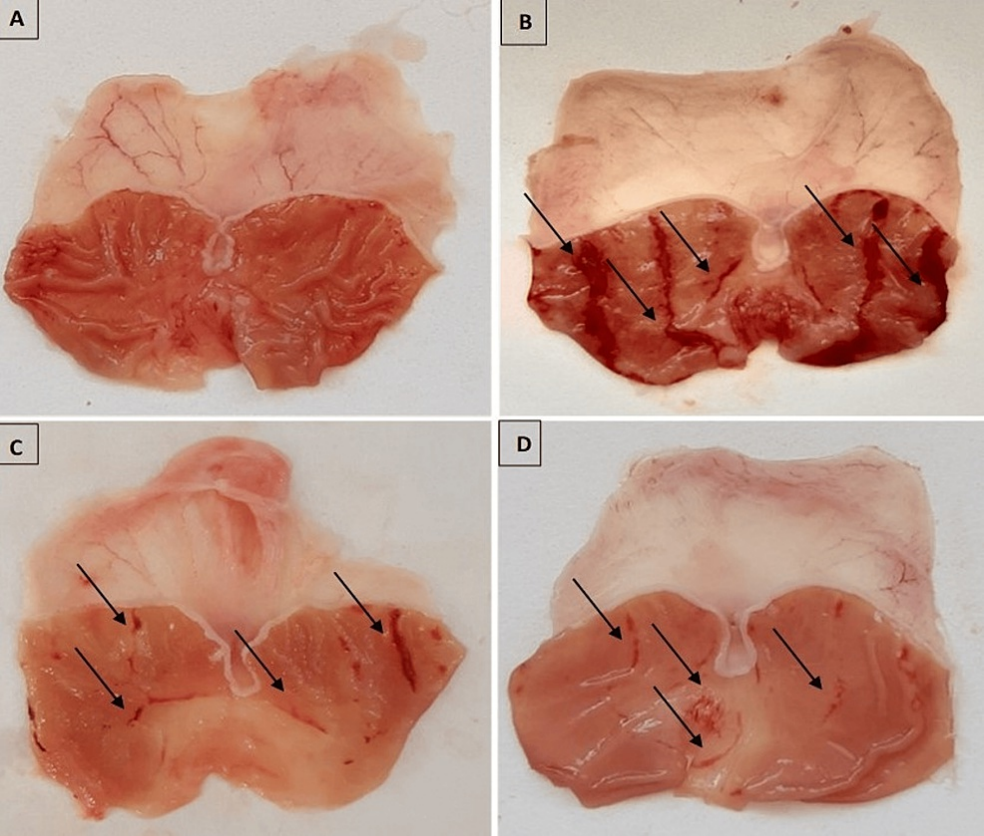
Influences of qaysum (AFE) on the macroscopic occurrence of gastric mucosa in ethanol-convinced stomach mucosa damage in rats. (A) In the negative control group, no macroscopic lesions were observed. (B) In the ulcer control group, rats receiving 96% ethanol developed a consistent pattern of visible damage and severe lesions with broad visible hemorrhagic ulceration at the gastric mucosa level. (C) Rats treated orally with the AFE (800 mg/kg) exhibited significantly reduced injuries on the mucosa of gastric in the animals. (D) The positive group (omeprazole + ethanol, 20 mg/kg) exhibited significantly reduced gastric lesions compared to the positive control group.

No macroscopic lesions were detected in the negative control group (G1) (Figure 2A). Gastric mucosal lesions were meaningfully reduced in animals pretreated with the AFE (G3) (Figure 2C). Omeprazole significantly reduced gastric lesions versus positive control (Figures 2B-2D), respectively.

Histological examination

**Figure 3:**
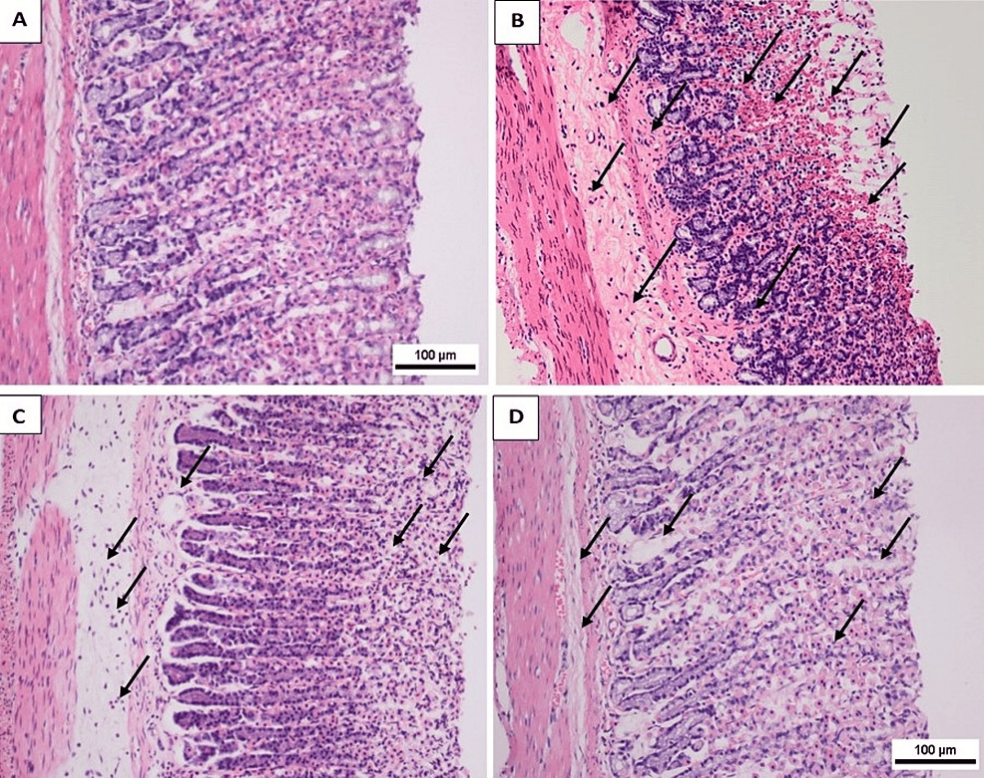
Influences of qaysum (AFE) on the microscopic expression of the gastric mucosa in ethanol-convinced gastric ulcers in rats. (A) The negative control group revealed a normal microscopic configuration of the gastric mucosa. (B) The ulcer control group appeared with severe formational damage of the gastric mucosa with edema and leucocyte entrance of the submucosal layer. (C) The group given Achillea fragrantissima showed temperate destruction to the gastric mucosa. (D) The omeprazole group exhibited mild damage to the mucosal layer of the gastric mucosa (H&E stain, magnification 10×).

Normal histological structure of surface lining epithelium, normal gastric epithelium with organized glandular structure, normal submucosa, and short gastric pits were observed in G1 (Figure 3A). Animals receiving 96% ethanol (G2) developed visible damage and severe injuries with wide visible hemorrhagic ulceration of stomach mucosa (Figure 3B). A photomicrograph of the gastric mucosa of (G2) showed sloughing of surface epithelial layer(s) and stomach pits, condensed mucosal thickness and slanted arrangement of glands in the mucosa, damage to gastric mucosa with observable hemorrhage and edema in the submucosa (Figure 3B). The gastric mucosa of (G2) showed exfoliated epithelial cells into the lumen, degeneration of surface epithelium (s) with deeply stained pyknotic nuclei, disrupted glandular structure, heavy leucocyte infiltration of lamina propria, vacuolization of some cells, congested blood vessels between gastric gland, edema of the submucosa, and inflammatory infiltrate (Figure 3B).

A photomicrograph of the gastric mucosa of (G3) showed protection of stomach mucosa, but the facile area of the stomach gland is minor slanted versus the ethanol-treated group. Extensive areas appeared in lamina propria among glands (Figure 3C). The gastric mucosa of (G3) showed lessening of surface epitheliums with denudation of gastric pits. Gastric glands showed inadequate restoration of their lining epithelium layer, less disruption to the gastric mucosa layer, reorganized glandular structure, and improvement of edema, but mild leucocyte infiltration was observed (Figure 3C).

A photomicrograph of the gastric mucosa of G4 showed a complete surface epithelial area with preserved glandular construction and surface mucous appearance with vacuolated cytoplasm cells. Marked improvement in gastric mucosa compared with all previously treated groups. The histological structure of the gastric gland appears similar to control with mild leukocyte penetration and edema (Figure 3D).

AFE effects on the PAS stain

**Figure 4:**
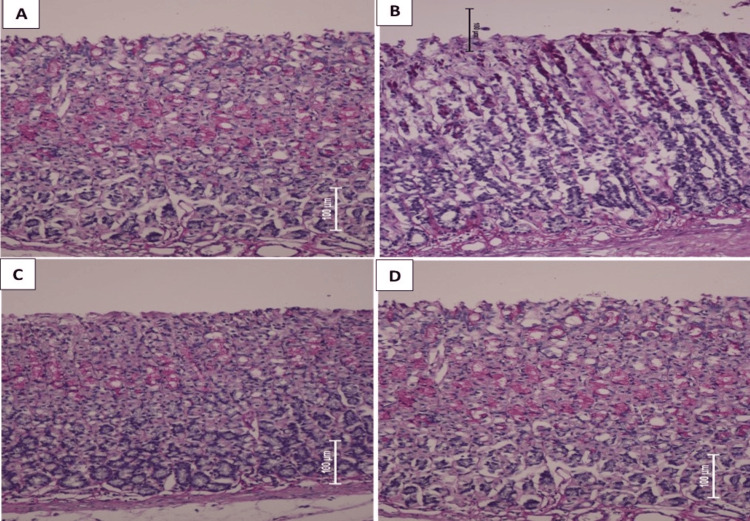
Influence of qaysum (AFE) on histological remark PAS glycoprotein stained (A-D). PAS-stained stomach epithelia in ethanol-convinced stomach ulcer. (A) Normal assemblage, usual stomach epithelial construction, as well as no symbols injury. (B) Ulcerated collection and severe destruction of stomach epithelia besides slight PAS stains. (C) Treated Achillea fragrantissima group, with slight destruction of stomach epithelia, with temperate PAS staining the stomach epithelia. (D) Omeprazole group, unimportant injury; exhaustive PAS stains the stomach epithelia (PAS stains amplification 20x).

The PAS staining method provides a quantity of the polysaccharides of mucus, demonstrated by the greater color intensity in the trial and negative control (Figure 4A) groups. Positive group rats (Figure 4B) had a minor intensity of PAS in their stomach mucosa than that of pre-treated rats. The PAS appearances were the same and analogous among omeprazole (Figure 4D) and AFE-treated rats (Figure 4C). The result specified that the AFE has the potential to up-normalize the discharge of stomach mucus, which performs as a guard fence against ethanol-convinced stomach damage.

## Discussion

In this current research, an oral acute toxicity experiment of the AFE on investigational animals revealed that there was no death for the period of the test in rats nourished with AFE compared to the negative control group, verifying the inoffensive plant extract. Congruently, numerous pieces of training by diverse academics using various plant excerpts have revealed its harmlessness, and no signs of poisonousness were observed [5,13]. GU is the most prevalent illness, with a frequency of 20-60 per 100,000 people and a death rate of 5-10% globally [14]. Many antiulcer medications, such as antacids, misoprostol, and PPIs, are used to prevent and treat stomach boils; however, these medications have many side effects. As a result, there is growing interest in herbal therapy to prevent or treat acute and chronic gastrointestinal diseases: by increasing antioxidant activity, preventing *H. pylori* colonization, balancing prostaglandins and anti-angiogenic factor regulation, minimizing oxidative mucosal changes, enhancing nitric oxide synthase and derived nitric oxide in the epithelial tissue, and boosting endogenous mucosal nitric oxide [6].

The weight gains and percentage changes in weight gain were significantly increased in ethanol plus AFE, but were significantly decreased in the omeprazole group versus the negative control and positive control groups. In this respect, long-standing omeprazole usage in young male rats decreased body weight gain [15].

In this study, ethanol led to increased gastric acidity and decreased stomach pH. Gastric ulceration by ethanol increased gastric volume and total acidity due to the released HCL secretion from the parietal cell surface [16]. Meanwhile, this study revealed that administration of AFE or omeprazole before ethanol led to a decline in gastric acidity and increased gastric pH. Stomach protection with the administration of ethyl acetate extract (EAE) of *A. biebersteinii *(200 mg/kg) before one week of GU stimulation. They documented a significant decline in stomach volume by 45.11%, total gastric acidity by 38.73%, and ulcer counts diminished by 47.05% versus the ulcerative group [16].

One of a vast number of substituted benzimidazoles, omeprazole, was created as a peptic ulcer medication as an inhibitor of acid secretion. Gaoet al.reported that omeprazole (15 mg/kg) medication for 14 days suppresses gastric acid production, greatly limits the amount of H+ that diffuses from gastric parietal cells to the mucosa, and raises gastric pH [17].

The current study showed that the UI increased in ethanol and AFE + ethanol group, versus the negative control group. Meanwhile, the UI significantly decreased in the omeprazole + ethanol groups versus the ethanol group. This study also revealed that the protective index in AFE + ethanol and omeprazole + ethanol were 20.95 and 98.02, respectively. These results indicated the protective effects of omeprazole administration against gastric ulcers. Abdel-Rahman et al. reported that AFE administration has an anti-ulcerogenic effect on stomach ulcers induced by pylorus ligation in rats [18].

The present study reported that oral ethanol administration led to a rise in MDA levels and a decline in CAT, GSH, and SOD levels in both serum and gastric homogenates. These findings were consistent with earlier studies [19] that observed a significant increase in MDA levels, indicating acute oxidative stress, contributing to ethanol-induced gastric injury development. Several researchers reported significant elevation in MDA levels in ethanol-induced gastric ulcers in rat models, reflecting free radical-mediated gastric mucosal damage [16]. MDA causes damage to skin fluidity, blight ion conveyance, and cellular utilities [20]. The CAT level decline observed in this study could cause an increased flux of superoxide radicals that increase lipid peroxidation and oxidative stress rate [12]. Increased actions of GSH peroxidase and GSH transferase (enzymes responsible for toxic compounds conjugation with GSH) primes to reduced GSH concentration.

In agreement with the results of this study, others [12,21] reported that AFE administration decreased MDA levels but increased CAT, GSH, and SOD levels compared to the ethanol-only group. Alhomaid et al. reported that serum CAT and SOD levels significantly increased with the administration of a low dose of *A. fragrantissima* (300 mg/kg), whereas high dose administration (500 mg/kg) had a mild effect [21]. Abd EL-Fattah et al. reported that alcoholic and ethyl acetate extracts of *A. fragrantissima* administration in type 2 diabetes mellitus rats exhibited activity against oxidative stress parameters due to the presence of various phenolic compounds [22].

In the present research, AFE, as well as the administration of omeprazole, also showed a significant decline in MDA but significantly increased CAT, GSH, and SOD levels in the serum and gastric homogenates versus the positive control group. The study reported important antioxidant effects of omeprazole along with its acid-suppression activities [23].

The role of inflammatory cytokines in stomach ulcer pathogenesis has been recognized previously [22,24]. This study agrees with all previous findings that implicate TNF-α level is remarkably augmented in serum and stomach tissue of ethanol-influenced ulcers [25]. Inflammatory cells that invade the body may be a significant source of ROS and consequent oxidative stress. Consequently, the gastric mucosa develops a spiteful rotation of inflammation and unnecessary free drastic formation, undermining the body's natural antioxidant defenses. According to the results of this study, pretreatment with AFE + ethanol significantly reduced TNF-α levels in serum and gastric homogenates versus the ethanol group. As a result, TNF-α inhibition in gastric tissue must be a vital mechanism contributing to the protective influence of these plant extracts against ethanol-convinced gastric injuries. AFE's ability to reduce mucosal inflammation in the stomach may account for its mechanism of achievement in diminishing stomach mucosal damage. Researchers attributed AFE's anti-inflammatory effect to the decline of cytokines, such as IL-1, IL-6, and TNF-α [5,16]. Beserra et al. reported that rats subjected to ethanol-induced ulcers after treatment with ellagic acid (10 mg/kg) showed significantly lower TNF-α levels versus the ethanol group [26].

Omeprazole also decreased TNF-α levels; this indicates that adequate inhibition of stomach acidity by omeprazole repressed inflammatory reactions, which advised that acid may well improve mucosal inflammation layers in response to pro-inflammatory cytokines stimulus, subsequent in gastric ulcers [27].

In this study, the histological investigation of the stomach revealed that, in the positive control group, the gastric mucosa layer showed severe lesions, including gastric pits, loss of surface epithelial layer, slight edema, and hemorrhage in the submucosa. Other histo-architecture sections expressed some leucocyte infiltrations scattered in gastric mucosa, vacuolization, and brushing off epithelial cells into the glandular lumen. An investigation observed that rats from the ethanol-administered group had severe ulcers with elongated-band bleeding in the stomach glandular layer [28]. These histopathological damages may be due to increased oxidative stress and lipid peroxidation by ethanol administration.

Our outcome exposed that investigational rats nourished with the AFE presented an augmented intensity of PAS discoloration in stomach segments in contrast to the positive control cluster. By the same token, recent researchers using dissimilar pharmaceutical plants have described an augmented strength of PAS pigmentation in gastric slices of investigational rats [29]. Pretreatment with AFE expressively improved the stomach mucus-making and reduced the acid pH of the stomach content. The PAS stain results showed an upsurge in mucus secretion in the stomach walls of rats pretreated with the AFE, suggestive of the gastro-protective act of AFE on the strength power of mucus liberation. These data prop the discovery of other studies that have an upsurge in gastric mucus concentration of PAS discoloration in rodents pre-fed with various plant extracts against necrotizing mediators to boost stomach damage of mucosal layers [4,5]. PAS dyes reduced by ethanol were improved by AFE pre-treatment, which also upsurged the glycoprotein content [29]. ROS, as a superoxide radical anion, is created through polymorphonuclear neutrophils, resulting in a reply from the cellular lipids and the manufacture of lipid peroxides. The main pointer of oxidative stress causing mucosal destruction could be MDA, which is a principal fat peroxidation metabolite [1].

The AFE at a dosage of 800 mg/kg, followed by ethanol (1 mL/kg), was given in this study. The photomicrograph of gastric mucosa after AFE + ethanol treatments showed marked restoration of the gastric mucosa and slight distortion of gastric glands of the surface region. AFE and ethanol treatments also attenuated superficial epithelium with denudation of gastric pits. The fundus glands were partially restored gastric epithelium, mucosal layer, mild leucocyte infiltration, and edema. Taking *Achillea* species extract orally helped prevent acute stomach lesions brought on by aspirin [30]. An earlier study on ethanol-induced GU and their amelioration by *Achillea* species extract reduced the oxidative stress and ulcer index in rats [16]. These investigations depicted the remarkable antiulcer effect of *Achillea *species.

Limitation of study

This study faced several limitations due to the difficulty of collecting plants, preparing plant extracts, and raising mice. The first limitation of this study was the scarcity of qaysum in the study area, and it is a seasonal plant; this made it difficult to collect a large enough sample, which led to a delay in the research process.

The second limitation is that the sample size is very small. There were only 28 rats being used. They were divided into four groups, including the control groups. This will increase the probability of getting false-positive and false-negative results. If we increase the sample size, we may lower the chance of error and can create more studied groups.

Third, there were only two different doses that have been studied. The comparison of these two doses is not enough to determine the optimum effective doses for preventing GU. The result obtained previously only shows a rough concentration that probably may contribute to the antiulcer activity. Thus, it gives a rough idea of what concentration could be used in preventing ulcers.

Fourth, in the histopathological examination, another staining method could be used to see the variability in the compound microscope. Alcian blue and Feulgen stain are some of the stains that can be used instead of H&E stain.

## Conclusions

The current study indicates that the AFE has a protective antiulcer efficacy in ethanol-induced peptic ulcers in rat models by decreasing inflammation and oxidative stress marker expression. This study introduced the AFE as an effective and economical antiulcer compound. Inappropriately, no clinical scholarships coping with the anti-inflammatory bustle of the AFE at the stomach flat have been instituted, therefore signifying that the influence of excerpts and distinct composites in this region needs to be clarified. Especially, it is essential to entice clinical hearings bearing in mind AFE belongings in patients with *H. pylori*-convinced inflammation of the stomach, unaccompanied or in a mixture with antibiotics.

## Figures and Tables

**Figure 5:**
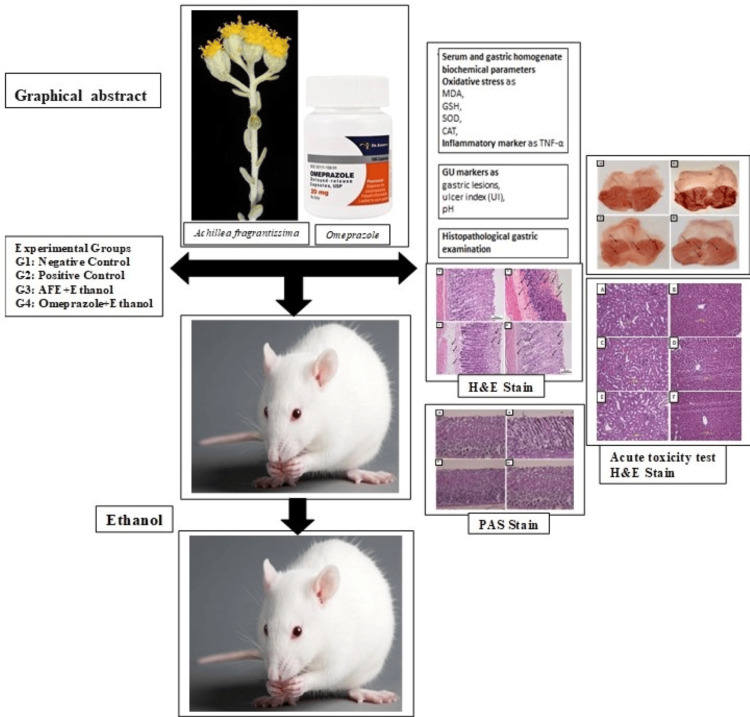
Graphical Abstract
